# Global health aid allocation in the 21st century

**DOI:** 10.1093/heapol/czx193

**Published:** 2018-02-05

**Authors:** Jesse B Bump

**Affiliations:** Department of Global Health and Population, Harvard TH Chan School of Public Health, 677 Huntington Ave, Boston, MA 02115, USA

The ways multilateral agencies allocate support are idiosyncratic, include opaque judgments made with undisclosed criteria, and lead to results that are not widely disclosed. This presents deep challenges for accountability and legitimacy, and raises serious questions about how well the needs of recipient countries are assessed and addressed. The stakes are very high, and the underlying issues are very important. These include how agencies define need, determine eligibility, and decide what support to provide to whom. The governance of these processes is also crucial. However, allocation has attracted very little scrutiny. The present special issue, *Beyond Gross National Income: Innovative methods for global health aid allocation*, represents the efforts of independent academics to bring attention to allocation processes and provide ideas and insights to improve debate around it.

The consequences of aid allocation decisions are enormous, and are felt most directly by the six billion people living in low- and middle-income countries (LMICs). For instance, these processes channel most of the medicines benefitting the 20.9 million people who accessed HIV therapies in 2017, and also played a role in not supporting treatments for the remaining approximately 16 million people living with HIV (UNAIDS 2017). These processes also shape many other aspects of the health services and interventions available to poor people in LMICs, such as in the emphasis on malaria, tuberculosis, and vaccine preventable diseases, and in the comparative indifference to non-communicable diseases, mental health or urban sanitation and other broad areas with tremendous influence on health. The significance of these decisions is heightened by the sheer quantity of money flowing through the system. In total, development assistance for health amounted to $36.4 billion in 2015; a roughly similar amount was provided in each of the previous five years, as well (Dieleman et al. 2016).

We believe this discussion is particularly timely because many multilaterals are struggling to address a major limitation at the center of most allocation processes. Economic performance estimates are not very reliable in many low- and middle-income countries (LMICs) but they have long been used by multilaterals as a primary criterion for aid eligibility. The fundamental flaw in this practice was exposed prominently in late 2010 when Ghana updated the base year in its gross domestic product (GDP) calculation, pushing up the (estimated) size of its economy by 69% from one day to the next (Moss and Majerowicz 2012). Whatever needs Ghana may have had did not change, and public discourse immediately erupted over the prospect of Ghana losing access to international assistance if its on paper GDP were estimated too high (Jerven 2012). Similar concern accompanied recalculation elsewhere, as when GDP in Nigeria, Kenya and Tanzania was estimated as 89%, 24% and 32% higher, respectively in 2013 and 2014 (Manson 2014, UNDP 2014, Blas and Wallis 2013). These and other similarly dramatic changes in estimated economic performance raised doubts about whether GDP could be usefully employed as an indication of need for assistance in health or other areas.

The importance of allocation processes demands transparency, and the sensitivity of GDP estimation points to the necessity of establishing a more reliable indicator of need. However, for the most part allocation processes remain hidden from public view, with little or no information available on the values they embody, the exact criteria they include, the quantitative and qualitative considerations they use, or the results they produce. With only a handful of exceptions, scholars have neglected this area, as well. Kanbur (2005) and Leo (2010) have examined the allocation formula at the World Bank’s International Development Association. Sridhar and Batniji (2008) have called on donors to disclose their disbursements. Fan et al. (2014) have analysed allocation under the Global Fund’s New Funding Model, and Ottersen et al. (2017) studied the criteria used by donors to make allocation decisions. Hanlon et al. (2014) show that the distribution of aid does not correspond well to disease burdens. A much larger literature has examined the politics of certain issues or the politics of donor investments (Woods 2005, Dunning 2004, Shiffman and Smith 2007, Geneau et al. 2010, Hafner and Shiffman 2013) but does not illuminate the decision making process as broadly or clearly as can a focus on allocation, even though these have been very valuable contributions toward other objectives.

The articles presented here represent a shared conviction that public discussion is an essential element in the legitimacy of these processes and that scholarly examination can contribute new ideas to make them more equitable, more efficient and more able to meet the needs of LMICs and their citizens. The ideas we explore here first took shape as part of the conversation begun by the Equitable Access Initiative (EAI), which was convened in 2015–2016 by Gavi, the Vaccine Alliance; the Global Fund to Fight AIDS, TB and Malaria; UNAIDS; the United Nations Development Programme; the United Nations Population Fund; UNITAID; UNICEF; the World Bank; and the World Health Organization. The convening agencies were motivated to explore classification schemes because in the past many have relied heavily on Gross National Income per capita (GNIpc) as a primary indicator of need and capacity, but there are now many reasons to revisit this practice. As noted in the EAI final report (EAI 2016, p. 4)

‘…the largest share of disease burden is now concentrated in middle-income rather than low-income countries, a reality that GNI per capita alone cannot capture. As a result, there is an increasing concern over the potential mismatch between GNI per capita and the extent of a country’s health needs suggesting that policies based on income classification alone overlook important dimensions of development, such as poverty and inequality.’

The EAI convened academic teams to explore related questions and although some ideas can be traced to that beginning, the articles we publish here represent the views of the authors alone and were developed independently after the EAI ended. In this connection, we gratefully acknowledge the support of the Wellcome Trust [099114/Z/12/Z]. We are also grateful to the Rockefeller Foundation for support of a meeting we convened at their Bellagio facilities to enhance our thinking on allocation and refine the arguments made here. All views remain our own.

In this special issue, we follow a sequence with our five articles, focusing in turn on how allocation works now, testing the correlation of GNIpc and health outcomes, exploring the normative basis for allocation through stakeholder preferences, analyzing how those preferences would change allocation rankings, and concluding with a model for calculating financing gaps to show where aid can make the most difference. Chi and Bump analyse current allocation practices at nine multilateral organizations with significant activities in health to better understand how allocation happens, examine some of the values behind various choices, and analyse what is included in the decisions to determine who gets what. They find that none of the processes could be understood based on public information alone, all are sensitive to non-disclosed adjustments, and very few included significant engagement with recipient countries. This comprehensive account of allocation processes is the first of its kind. Sterck et al. turn to an empirical investigation to analyse whether GNIpc holds substantial explanatory power for health outcomes. It does not. This finding shows that the technical basis for using GNIpc as a leading element in allocation is actually very weak. Grépin et al. use a survey and discrete choice experiment to gather stakeholder views on the values that should guide allocation. They find that respondents emphasize burden of disease and health inequalities as far more important characteristics for allocation than GNIpc. This means that global health professionals have great reservations about the ethical basis for using GNIpc. In a related exercise, Ottersen et al. developed classification frameworks that were used to demonstrate the effect of including other indicators along with GNI per capita. They find that integrating a health needs indicator substantially changes the ranking of countries compared with using GNIpc alone. This means that if allocation reflected the actual values of global health professionals, it would differ greatly from allocation based on GNIpc. In the final paper, Haakenstad and colleagues propose a fundamental reorientation of allocation from measures of need to quantification of opportunities. By estimating the health gains that could be made via spending in various areas, their framework helps policymakers compare the consequences of different investment decisions.

There are many countries for which health assistance allocation is a paramount concern. Many of these countries are in transition. Donors and agencies have used this term to describe the reduction and eventual cessation of assistance as countries become wealthier. Quite reasonably donors want to guide resources to where needed most, but virtually all allocation is still centered on GNI. The articles in this special issue show how profoundly problematic this practice has become from both technical and ethical perspectives. Our work provides the basis for countries to contest and shape these decisions by explicating current allocation practices, clarifying many of the choices surrounding them, and proposing improved alternatives. We hope to improve the legitimacy, responsiveness and efficacy of development assistance by fostering more open discussion and more transparency. However, we note with caution the limits of such a conversation if conducted by technical experts and multilaterals alone. Of all the problems in current allocation, the very low inclusion of recipient countries and citizens is the most glaring. The broader goals of advancing development and promoting more effective collaboration between countries include choices that can be made legitimate only through systematic efforts to enfranchize those who ostensibly benefit. As a step toward that, we offer these articles to start discussion among a much broader, more inclusive audience. We look forward to the conversation.

## References

[czx193-B1] Blas J, Wallis W. 2013 *Nigeria almost doubles GDP in recalculation* *Financial Times* https://www.ft.com/content/70b594fe-bd94-11e3-a5ba-00144feabdc0, accessed 7 April 2013.

[czx193-B2] Dieleman JL, Schneider MT, Haakenstad A 2016 Development assistance for health: past trends, associations, and the future of international financial flows for health. The Lancet 387: 2536–44. 10.1016/S0140-6736(16)30168-4 27086170

[czx193-B3] Dunning T. 2004 Conditioning the effects of aid: Cold War politics, donor credibility, and democracy in Africa. International Organization 58: 409–23.

[czx193-B4] EAI, 2016 *The Equitable Access Initiative Final Report* https://www.theglobalfund.org/media/1322/eai_equitableaccessinitiative_report_en.pdf?u=636488964380000000.

[czx193-B5] Fan VY, Glassman A, Silverman RL. 2014 How a new funding model will shift allocations from the Global Fund to Fight AIDS, tuberculosis, and malaria. Health Affairs 33: 2238–46. 25392001 10.1377/hlthaff.2014.0240

[czx193-B6] Hafner T, Shiffman J. 2013 The emergence of global attention to health systems strengthening. Health Policy and Planning 28: 41–50. 22407017 10.1093/heapol/czs023

[czx193-B7] Hanlon M, Graves CM, Brooks BP 2014 Regional variation in the allocation of development assistance for health. Globalization and Health 10: 8. 24555735 10.1186/1744-8603-10-8 PMC3936884

[czx193-B8] Jerven M. 2012 *Lies, damn lies and GDP. The Guardian.* https://www.theguardian.com/business/2012/nov/20/economics-ghana, accessed 20 November 2012.

[czx193-B9] Kanbur R. 2005 *Reforming the Formula: A Modest Proposal for Introducing Development Outcomes in IDA Allocation Procedures. CEPR Discussion Paper No. 4971* SSRN: https://ssrn.com/abstract=771636.

[czx193-B10] Geneau R, Stuckler D, Stachenko S 2010 Raising the priority of preventing chronic diseases: a political process. The Lancet 376: 1689–98. 10.1016/S0140-6736(10)61414-6 21074260

[czx193-B11] Leo B. 2010 *Inside the World Bank’s Black Box Allocation System: How Well Does IDA Allocate Resources to the Neediest and Most Vulnerable Countries?* SSRN: https://ssrn.com/abstract=1646613.

[czx193-B12] Manson K. 2014 *Tanzania joins Africa’s great GDP revision. Financial Times.* https://www.ft.com/content/485f956d-6673-3f15-856a-06a9a7028481, accessed 19 December 2014.

[czx193-B13] Moss TJ, Majerowicz S. 2012 *No Longer Poor: Ghana’s New Income Status and Implications of Graduation from IDA* Center for Global Development Working Paper No. 300. SSRN: https://ssrn.com/abstract=2102796.

[czx193-B14] Ottersen T, Kamath A, Moon S, Martinsen L, Røttingen JA. 2017 Development assistance for health: what criteria do multi-and bilateral funders use?. Health Economics, Policy and Law 12: 223–44. 10.1017/S1744133116000475 28332462

[czx193-B15] Shiffman J, Smith S. 2007 Generation of political priority for global health initiatives: a framework and case study of maternal mortality. The Lancet 370: 1370–9. 10.1016/S0140-6736(07)61579-7 17933652

[czx193-B16] Sridhar D, Batniji R. 2008 Misfinancing global health: a case for transparency in disbursements and decision making. The Lancet 372: 1185–91. 10.1016/S0140-6736(08)61485-3 18926279

[czx193-B17] UNAIDS, 2017 Fact Sheet: World AIDS Day 2017. Geneva: UNAIDS http://www.unaids.org/sites/default/files/media_asset/UNAIDS_FactSheet_en.pdf.

[czx193-B18] UNDP, 2014 Analysis of Likely Implications of Rebasing the GDP of Kenya. New York: United Nations Development Programme http://www.ke.undp.org/content/dam/kenya/docs/SPAU/Analysis%20of%20Likely%20Implications%20of%20Rebasing%20the%20GDP%20of%20Kenya%20-%20Final%20Copy.pdf? download.

[czx193-B19] Woods N. 2005 The shifting politics of foreign aid. International Affairs 81: 393–409.

